# 3D printing improves preoperative decision making for patient positioning and surgical approach selection for tibial plateau fractures

**DOI:** 10.1038/s41598-025-11995-8

**Published:** 2025-07-22

**Authors:** Tobias Dust, Julian-Elias Henneberg, Maximilian J. Hartel, Alexander Korthaus, Tobias Ballhause, Fidelius von Rehlingen-Prinz, Anna Streckenbach, Johannes Keller, Karl-Heinz Frosch, Matthias Krause

**Affiliations:** 1 Department of Trauma and Orthopaedic Surgery, University Medical Center Hamburg – Eppendorf, Martinistraße 52, 20246 Hamburg, Germany; 2 Department of Trauma Surgery, Orthopaedics and Sports Traumatology, BG Hospital Hamburg, Hamburg, Germany; 3 Department of Diagnostic and Interventional Radiology and Nuclear Medicine, University Medical Center Hamburg-Eppendorf, Hamburg, Germany

**Keywords:** Tibial plateau fracture, 3D printing, Preoperative management, Surgical approach, Patient positioning, Implant selection, Three-dimensional imaging, Fracture repair, Trauma, Bone imaging, Computed tomography, Reconstruction

## Abstract

Treatment of complex tibial plateau fractures remains a challenging task in clinical practice. Sufficient and appropriate preoperative decision making is essential for optimal treatment success and ultimately influences patient outcomes. Recently, the novel technique of 3D printing has proven to be beneficial for the preoperative management in other joint regions. To investigate the impact of point-of-care 3D printing on the preoperative management of tibial plateau fractures, we asked 5 students, 10 surgical residents, 3 junior surgeons and 4 senior surgeons, to simulate the preoperative planning of 22 tibial plateau fractures (11 AO B and 11 AO C fractures) regarding the treatment concept, patient positioning, operative approach and implant selection and positioning. First with CT scans only, second with 3D volumetric reconstructions, and finally with 3D printed fracture models. We analyzed the inter- and intraobserver agreement and the subjective perceived confidence of the rater regarding his decision with the different imaging modalities across the different levels of professional experience. Statistics were performed using kappa values, percentage match (PM) analysis and a univariate one-way analysis of variance. The use of 3D printing had no effect on the interobserver reliability of treatment concept selection (PM CT 83% > 3DCT 83% > 3D 82%). However, descriptively higher kappa and percentage match values increased for agreement on patient positioning and surgical approach using 3D printed fracture models. In addition, the raters selected the implants that were actually used to treat the fractures in 63% of the cases. The subjective perceived certainty of the raters increased with the use of 3D printing technology from 45% (CT and 3DCT) to 60% (3D). Additionally, raters changed their treatment plan in 36% of the cases and gained additional information 76% of the time when using the 3D printed specimen. The use of 3D printed fracture models showed a trend toward higher interrater reliability of patient positioning and surgical approach for medical students and surgical residents, while experienced surgeons show less benefit. In addition, 3D-printed models supported implant pre-selection and increased subjective confidence, positively influencing preoperative planning.

## Introduction

The surgical treatment of complex tibial plateau fractures (TPF), especially bicondylar injuries, is one of the most challenging tasks in orthopaedic trauma surgery^1^. Despite technically complex diagnostics and intraoperative visualization with 3D-CT, postoperative malreduction is found in 32.3% of cases, especially in the area of the postero-lateral tibial plateau^2^. These malreductions can lead to clinically relevant leg axis malalignments resulting in pseudo-instabilities and ultimately secondary osteoarthritis^3^. Rates of posttraumatic osteoarthritis of up to 44% have been reported in the literature, especially for bicondylar fractures^4,5^. Moreover, only a minority of patients are able to return to their preinjury level of sports activity after tibial plateau fractures^6^. Therefore, the goal of surgical treatment of tibial plateau fractures is to reconstruct a near-anatomic articular surface, restore the physiologic leg axis, and address potential concomitant soft tissue injuries. Complete visualization of the entire fracture zone is an important factor for optimal articular surface reconstruction^7^. At the same time, the choice of surgical approach and appropriate patient positioning play a critical role in optimal fracture treatment and are still highly dependent on the surgeon’s experience^8,9^. This includes, but is not limited to, the choice of patient positioning, fixation materials, required surgical approaches and extensions as well as potentially required additional tools such as distractors, intraoperative 3D CT, or fracturoscopy, defined as the intraoperative use of arthroscopy to assist fracture reduction and evaluate the articular surface^7,10,11^. Traditionally, the majority of cases have been performed in the supine position^12^. However, as posterior segments of the lateral tibial plateau are frequently involved and often malreduced, alternative approaches in lateral or prone position have emerged and are associated with improved reduction outcomes^7,13–18^. In addition, optimal hardware positioning must be considered^19,20^. Bone quality and the potential use of bone graft substitutes must also be recognized^21–23^.

The aim of this study was to assess whether 3D-printed fracture models improve preoperative decision making for tibial plateau fractures compared to CT and 3DCT imaging, with a focus on patient positioning, surgical approach, and implant selection. We hypothesized that 3D printing would enhance inter- and intraobserver agreement and increase diagnostic confidence.

## Methods

Twenty-two adult cases with an intra-articular tibial plateau fracture(AO/OTA type B or C)^24^ treated surgically at our department between 2017 and 2020 were included. Inclusion criteria were: no history of previous proximal tibial fracture, and complete preoperative imaging documentation, consisting of digital anteroposterior and lateral radiographs of the knee, as well as CT scans with a maximum axial slice thickness of 1 mm and multiplanar reconstructions in the sagittal and coronal planes. Cases were selected consecutively from all eligible patients based on these inclusion criteria. The study was approved by the Ethics Committee of the Medical Chamber of Hamburg, Germany (ID 2024-101279-BO-ff) and conducted according to the guidelines of Good Clinical Practice and the recommendations of the Declaration of Helsinki.

### Image acquisition

CT scans of tibial plateau fractures were exported from the institution’s Picture Archiving and Communication System (PACS) and stored in DICOM (Digital Imaging and Communications in Medicine) format.

Three videos were generated from DICOM images (axial, sagittal, and coronal sequences of the CT scan) using Horos software (Horos Viewer for Mac, v. 3.3.6, Horos Project, USA). Each video consisted of 40 images and was implemented in the online survey tool as scrollable frames. This created a user interface (UI) was created similar to the in-house PACS system.

Using the same software package, two scrollable (transverse and longitudinal) 3D volumetric reconstructions of the proximal tibia and fibula with subtraction of the femur and patella consisting of 40 images were generated and implemented in an online survey tool.

### Segmentation

**Fig. 1:**
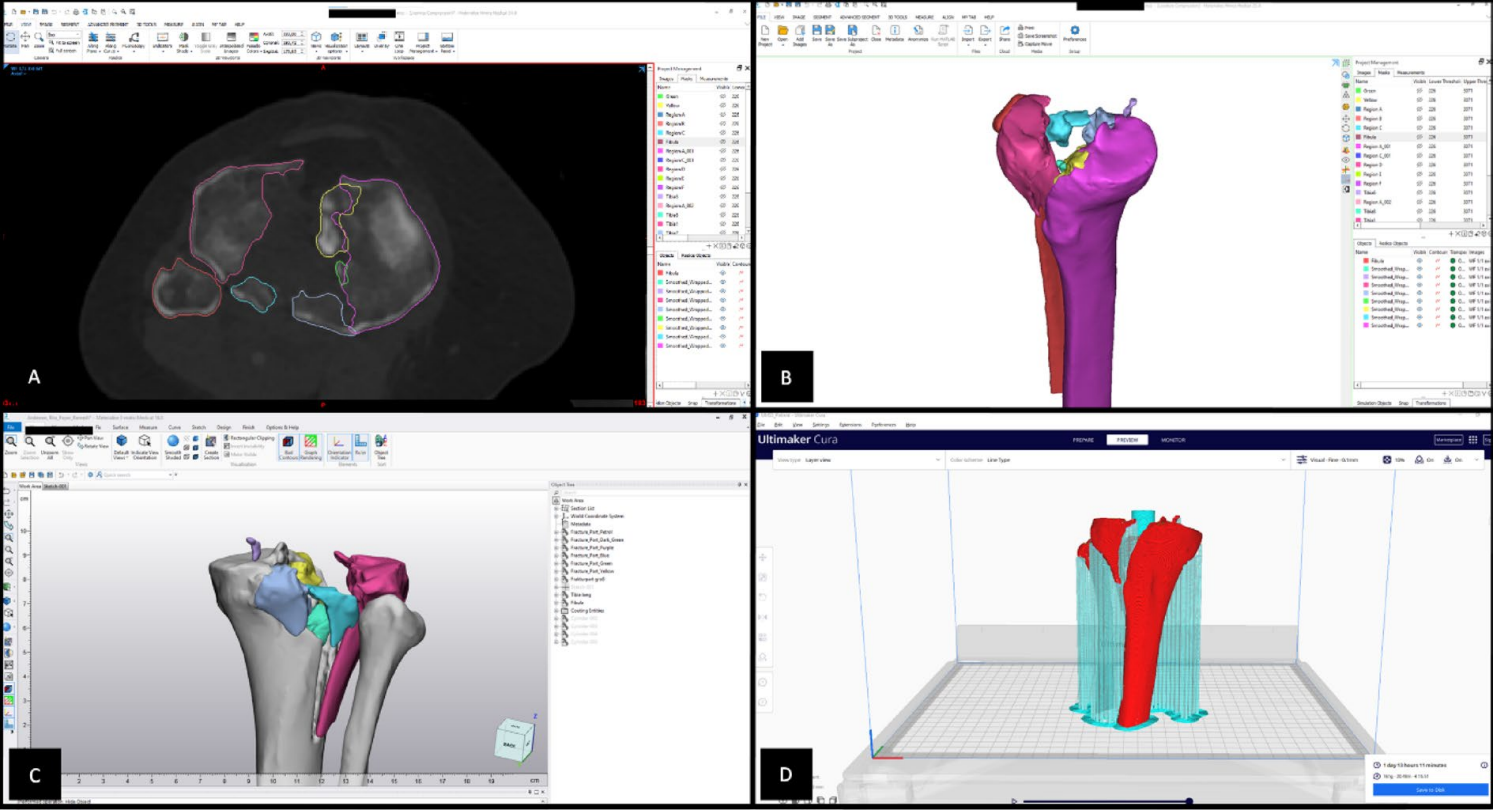
Threshold-based semi-automated segmentation using Materialise’s interactive medical image control system (Mimics Innovation Suite v24; Materialise, Leuven, Belgium) (**A,B**), post processing using Materialise 3-Matic (Materialise 3-Matic Medical v16; Materialise, Leuven, Belgium) (**C**), slicing process using Cura (Ultimaker Cura v4.10; Ultimaker, Utrecht, Netherlands) (**D**).

CT scans of these fractures were stored as complete DICOM series and processed using Materialise’s Interactive Medical Image Control System (Mimics Innovation Suite v24; Materialise, Leuven, Belgium (Fig. 1 (A), (B)). 3D reconstructions were generated using a threshold-based semi-automatic segmentation method with a threshold of 226 and higher to separate soft tissue and isolate bony structures. The femur and patella were digitally removed to improve visualization of the intra-articular fracture. Due to fragment depression and comminution, especially in complex, highly comminuted fracture types, some of the fragments had to be processed manually.

Segmented parts were exported to Materialise 3-Matic (Materialise 3-Matic Medical v16; Materialise, Leuven, Belgium; Fig. 1 (C)) and post-processed with global surface treatment and stabilization tubes for large fracture elements. 3D reconstructions were exported as Standard Tessellation Language (STL) files.

3D printing was performed using an Ultimaker S5 dual-head fused deposition modeling (FDM) printer. This printer is a high-end FDM printer with a large build volume and the ability to print simultaneously with two different print materials. Polylactic acid (PLA) was used to print the fracture model, water-soluble polyvinyl alcohol (PVA) served as the support material.

**Fig. 2:**
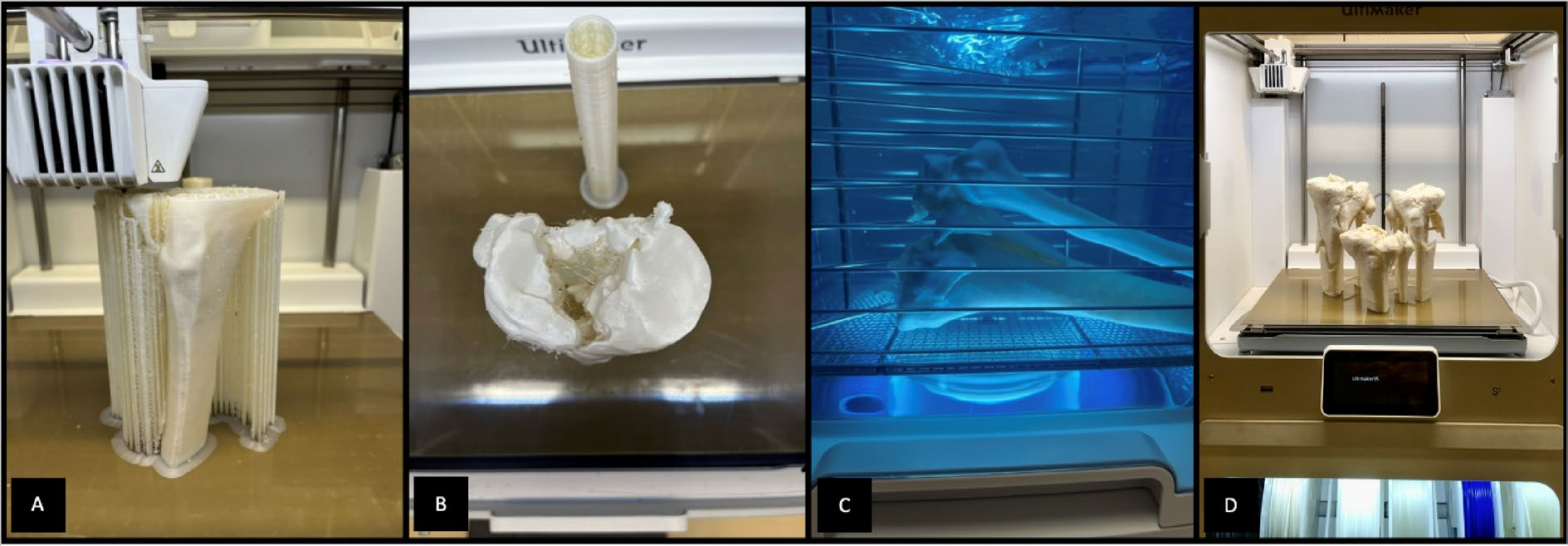
3D-printing station with a Ultimaker S5 FDM 3D printer (**A,B,D**) and a support dissolving station (**C**).

Cura (Ultimaker Cura v4.10; Ultimaker, Utrecht, Netherlands (Fig. 1 (D)) was utilized for the slicing process. The layer height was set to 0.1 mm to ensure a high level of detail. The STL file was converted to G-code to prepare the file for 3D printing. The tibial plateau fractures were printed at a scale of 1:1 (Fig. 2 (A, B, D)). After printing, the models required post-processing to remove the support structures and brim (Fig. 2 (C)).

### Online survey

**Fig. 3:**
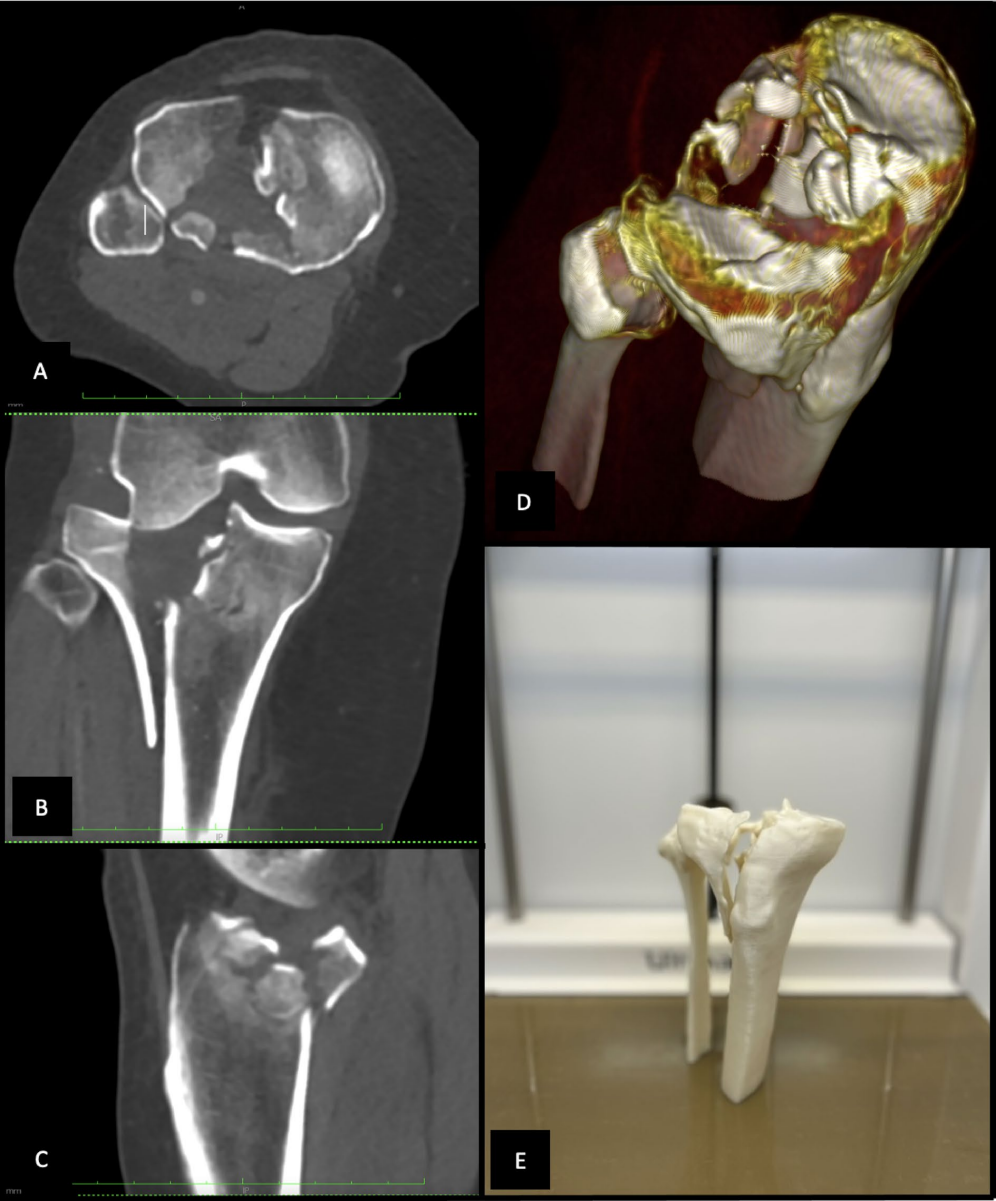
Multifragmentary lateral tibial plateau fracture shown as a CT-scan (**A–C**), a 3D volumetric reconstruction (**D**) and as a 3D-printed model (**E**).

An online survey was created using s2survey.net (SoSci Survey GmbH, Munich, Germany). A detailed set of instructions was sent to the observers in advance. For each patient, a case folder was created containing three scrollable interactive videos representing the axial, sagittal and coronal planes of the patient’s CT sequence, as well as two 3D volumetric reconstructions rotatable in the horizontal and vertical planes of the patients’ tibial plateau (Fig. 3A-C).

**Fig. 4:**
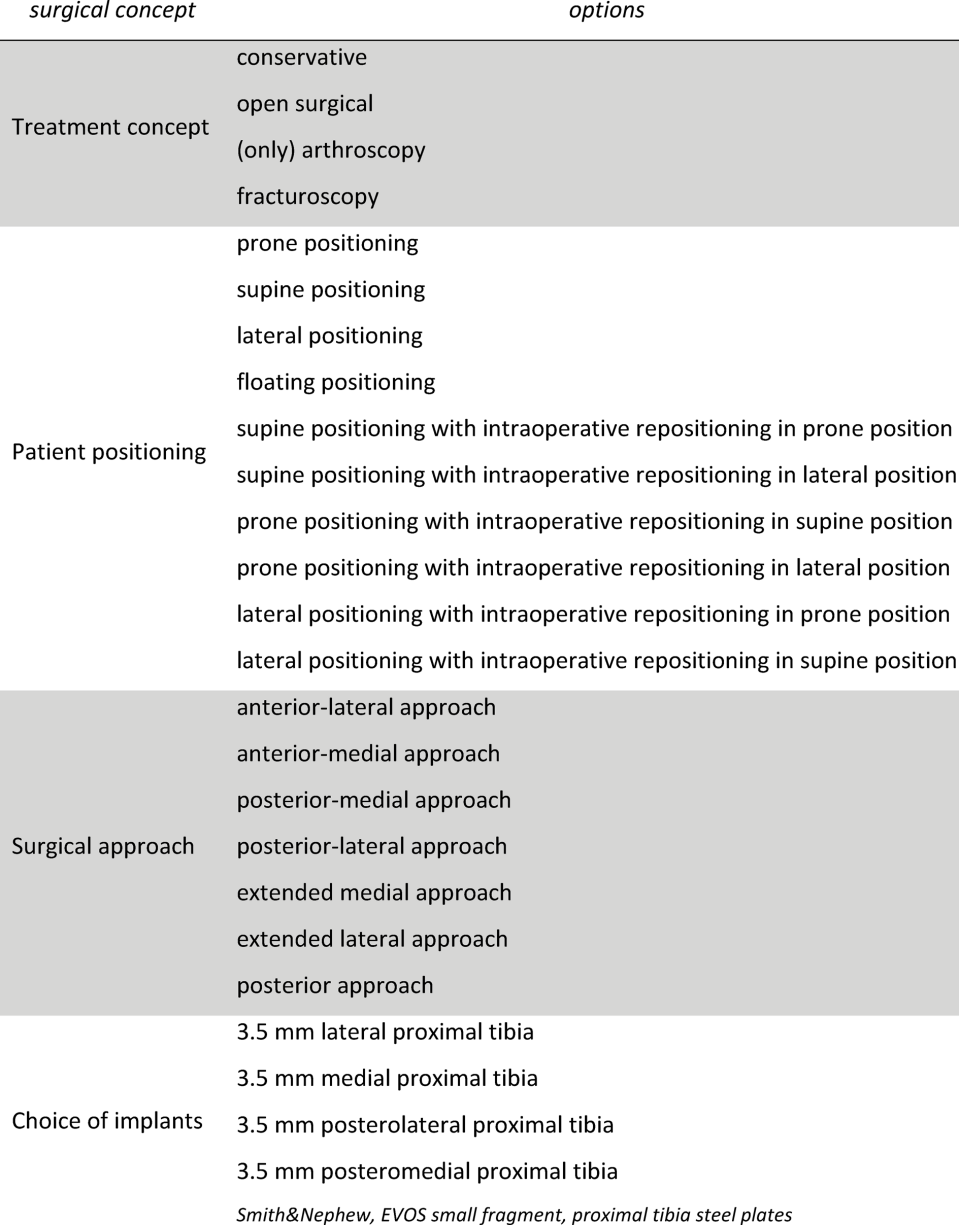
The different aspects and their options to choose from of the surgical concept.

**Fig. 5:**
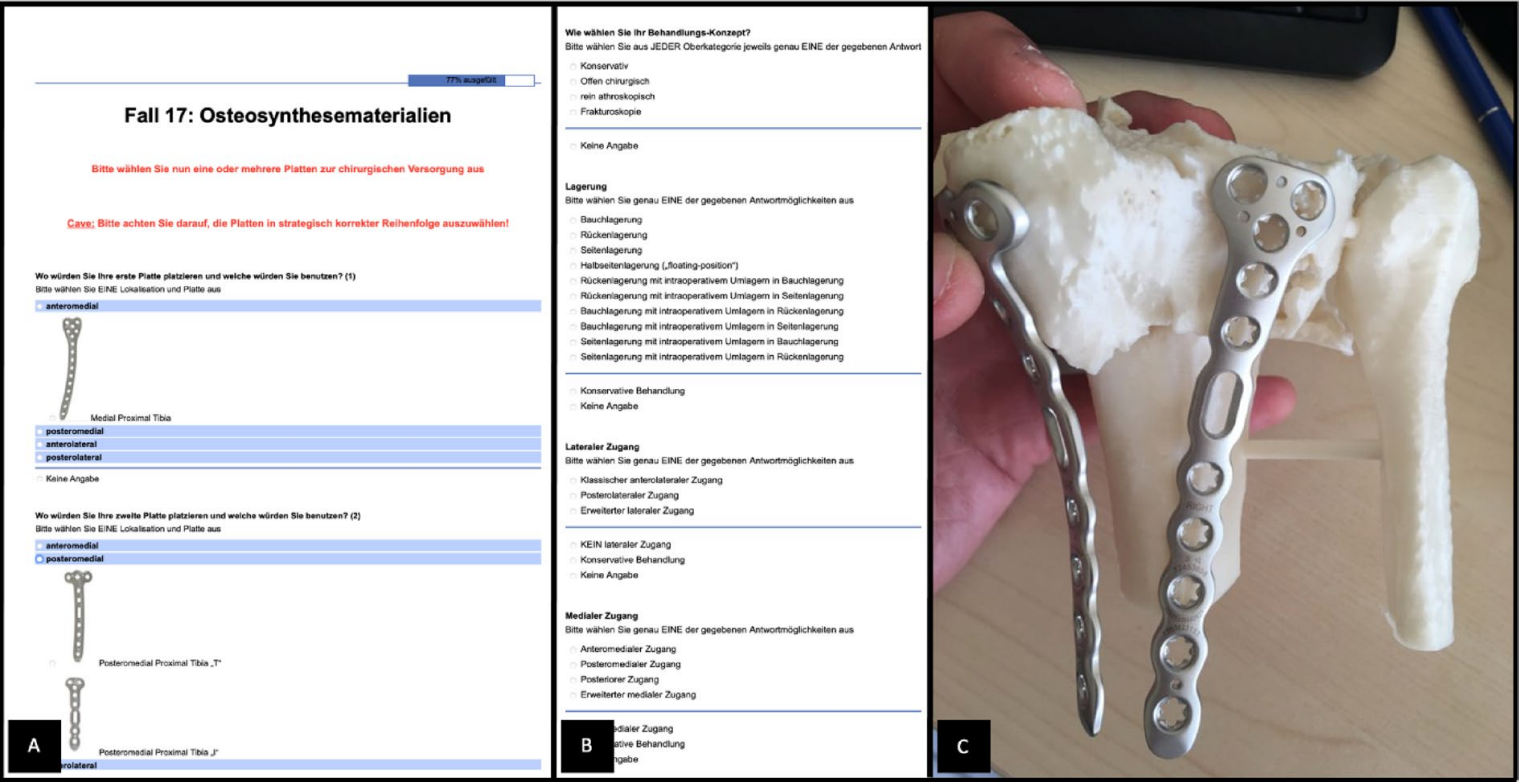
Online questionaire (**A,B**) and plate selection (**C**).

The folder consisted of three main pages: At the first page, the observers were shown traditional axial, coronal and sagittal CT images. At the second page, the observers were able to view 3D volumetric reconstructions (Fig. 3D). At the third page of the survey, observers were provided with the physical 3D-printed fracture models, allowing free handling and inspection from all angles (Fig. 3E). No digital images or photos of the printed models were used. Observers interacted directly with the models without supervision, following an initial standardized instruction session. At each decision-making stage, observers could select between conservative treatment, arthroscopy-only treatment, open surgery, or fracturoscopy (as previously defined). Although all fractures included in this study were ultimately treated surgically because of their displacement and complexity, conservative and arthroscopy-only options were intentionally included to capture the full theoretical decision spectrum during preoperative planning. Fracturoscopy was considered a variant of open surgical reduction, with the addition of arthroscopic assistance to improve visualization of the articular surface, reflecting a preference of some observers for enhanced intraoperative joint assessment. Each radiological dataset (CT, 3D volume reconstruction) was implemented in the survey as a scrollable hint to simulate a point-of-care image viewing station. At each stage, the raters were presented with the first three options to answer and the last page (3D) included the fourth question regarding treatment concept, patient position, choice of approach and choice of implant positioning (Figs. 4 and 5 (A, B)). Patient positioning options included supine, prone, lateral decubitus, floating positioning (initial supine positioning with intraoperative repositioning to prone or lateral positions if needed), and supine/prone/lateral positioning with planned intraoperative repositioning. The “floating” position referred to a non-fixed initial positioning strategy in which no definitive patient position was selected preoperatively. This option was intended to reflect a flexible surgical concept with intraoperative repositioning decided dynamically. In contrast, options such as “supine to prone” or “supine to lateral” represented predefined intraoperative changes of position based on a planned strategy. Surgical approach options included standard anterolateral, anteromedial, posteromedial, and posterolateral approaches, as well as extended medial and extended lateral approaches. Extended approaches were defined as enlarged exposures beyond standard approaches, such as lateral epicondyle osteotomy (for extended lateral access) or extensive posteromedial exposure, including medial epicondyle osteotomy (for extended medial access). Observers were allowed to select multiple surgical approaches if they considered a dual-approach strategy necessary^17^. A non-sterile plate system (EVOS, Smith and Nephew), routinely used at our institution for the surgical treatment of tibial plateau fractures, was provided to the raters for this step (Fig. 5 (C)). The type and size of osteosynthesis plates were determined on the 3D model and then compared with those used intraoperatively.

After each page, raters were asked whether they felt comfortable with their choices using a five-point Likert scale. It was possible to interrupt and resume at the same stage of the survey. However, it was not possible to return to a previous page and correct previous answers. After completion of the entire survey, the observers’ accounts were locked for four weeks. After that, the retests were carried out with the 22 cases.

In the 3D printing group, an additional yes/no question was presented after each individual case to evaluate whether the physical model had provided any added value beyond the imaging data. The question was phrased: “Did the 3D-printed model provide additional information relevant to your decision-making?” Responses were recorded as “Yes” or “No” for each case.

### Raters

The study included five medical students who had a strong interest in orthopaedic and trauma surgery and had completed at least one month of a clinical elective in this area. The ten surgical residents all had between one and six years of experience. The three junior surgeons and four senior surgeons were all orthopaedic and trauma surgeons with more than seven years of experience. To ensure valid intraobserver reliability testing, the number of retested observers and their distribution across experience levels were determined based on previously published relevant studies on fracture classification reliability^25–29^.

### Statistics

All responses were anonymously stored in an online database and then exported to Microsoft Excel (Microsoft Office 365, Microsoft Corporation, Redmond, USA). Descriptive statistics were used to analyze the parameters of the patient population, the printing process and to compare the selected implants. The Kappa coefficient was calculated and interpreted according to the criteria of Landis and Koch^30^ to analyze the inter- and intraobserver reliability of the treatment concept, patient positioning and surgical approach at each stage of the three steps^30–32^. Further percentage match evaluation (PM) was used to provide an additional measurement of the interobserver reliability to provide a descriptive metric that focused on the degree of agreement between raters’ decisions^33^. PM represents the proportion of identical ratings across raters, expressed as a percentage. While not an inferential statistic, PM is commonly used in applied research to provide an intuitive measure of agreement, particularly in contexts where kappa values may be affected by skewed response distributions or a high number of raters. In this study, PM was not intended to replace kappa, but to complement its interpretation and improve transparency in reporting interrater agreement. For both kappa and PM, 95% confidence intervals were computed. Five-point Likert Scales were used to quantify the subjective certainty of the rater’s decision. This was assessed using a standardized statement: “I feel confident in my decision regarding the overall treatment concept”. Responses were recorded on a 5-point Likert scale with the following options: “strongly disagree”, “disagree”, “neutral”, “agree”, and “strongly agree”. A univariate one-way analysis of variance (ANOVA) was used to determine whether there were significant differences between the groups (CT vs. 3DCT vs. 3D-print).

## Results

### Treatment concept

The overall reliability of the treatment concept showed a slightly higher (fair) reliability for fracture analysis using CT scans and 3D volumetric reconstructions. For 3D printed models, the kappa analysis showed a slight agreement (CT 0.23; 3DCT 0.21; 3D-print 0.17). Percentage match (PM) evaluation showed an agreement of 83% for CT and 3DCT, while the 3D print category reduced the PM to 82%. The statistics were extended to include PM because the kappa values showed the paradox of high agreement but low values due to the large number of raters and the influence of the prevalence of the evaluated entity. This phenomenon was described by Feinstein et al.^34^. To improve the visualization and comparability of the data, the statistics were supplemented with the PM (Table 1). The kappa values for intraobserver agreement showed the highest value in the 3D-printing category (Table 4).

**Table 1:** Interobserver agreements for the treatment concept.

Treatment concept	CT		3DCT		3D	
	PM (95% KI)	κ (95% KI)	PM (95% KI)	κ (95% KI)	PM (95% KI)	κ (95% KI)
Overall	83% (60–95%)	0.228 (0.227–0.228)	83% (60–95%)	0.209 (0.209–0.210)	82% (60–95%)	0.169 (0.168–0.170)
Medical students	93% (71–99%)	− 0.280 (− 0.31–− 0.25)	90% (71–99%)	0.004 (0.001–0.007)	88% (65–97%)	− 0.014 (− 0.018–− 0.011)
Surgical residents	79% (55–92%)	0.270 (0.268–0.271)	78% (55–92%)	0.206 (0.205–0.208)	78% (55–92%)	0.198 (0.196–0.199)
Junior surgeons	82% (60–95%)	0.177 (0.171–0.183)	82% (60–95%)	0.257 (0.251–0.263)	85% (65–97%)	0.299 (0.292–0.306)
Senior surgeons	83% (60–95%)	0.197 (0.193–0.201)	84% (60–95%)	0.067 (0.063–0.071)	83% (60–95%)	− 0.052 (− 0.056–− 0.048)

### Patient positioning

Regarding intraoperative patient positioning, kappa coefficient analysis showed overall a fair agreement. The overall κ value and PM improved from the use of CT scans alone to the use of 3D printed models. The medical students improved their PM and kappa value the most and made a kappa class switch from a slight to a fair agreement by using 3D printing (Table 2). Surgical residents also improved their values with 3D printing. However, the more experienced surgeons did not improve their values with 3D printing. The PM analysis showed similar trends. This indicates a benefit in decision making regarding patient positioning with the use of 3D printing, especially for inexperienced surgeons. The intraobserver agreement kappa analysis also showed moderate agreement (0.42) with the 3D-print showing the highest agreement (Table 4). In addition, in 21% of cases, the raters changed their patient positioning after evaluating the 3D print (Table 5). Overall, there was a slight trend toward repositioning the patient during the surgical procedure using 3D printing. The results did not reveal any discernible pattern with respect to patient positioning or any specific combination thereof. However, it is noteworthy that raters selected intraoperative repositioning in approximately 25% of cases across all modalities, despite the fact that all treatments were performed without patient repositioning. When comparing the selected patient positions for all modalities with those actually used to treat each fracture, 3D printing had the highest percentage match (54%) compared to CT (52%) and 3DCT (51%). Although improvements in both PM and κ values were observed, there was substantial overlap between the 95% confidence intervals across modalities and subgroups. Therefore, no statistically significant differences between CT, 3DCT, and 3D printing can be assumed.

**Table 2:** Interobserver agreements for the patient positioning.

Patient positioning	CT		3DCT		3D	
	PM (95% KI)	κ (95% KI)	PM (95% KI)	κ (95% KI)	PM (95% KI)	κ (95% KI)
Overall	46% (24–68%)	0.248 (0.247–0.248)	46% (24–68%)	0.264 (0.263–0.264)	53% (32–76%)	0.356 (0.356–0.356)
Medical students	35% (17–59%)	0.116 (0.114–0.118)	38% (17–59%)	0.154 (0.152–0.156)	55% (32–76%)	0.390 (0.388–0.392)
Surgical residents	43% (21–64%)	0.194 (0.192–0.195)	45% (24–68%)	0.224 (0.223–0.225)	51% (28–72%)	0.308 (0.307–0.309)
Junior surgeons	53% (32–76%)	0.321 (0.316–0.325)	52% (28–72%)	0.314 (0.310–0.318)	50% (28–72%)	0.302 (0.298–0.306)
Senior surgeons	65% (41–83%)	0.464 (0.460.–0.468)	61% (36–79%)	0.387 (0.383 –0.291)	61% (36–79%)	0.391 (0.388–0.395)

### Surgical approach

Medical students, surgical residents and senior surgeons were able to improve their kappa values and PM from CT to 3D printing. Junior surgeons decreased their scores (Table 3). The Intraobserver analysis showed an overall fair agreement (0.37) with the 3D print again having the highest value (Table 4). This again highlights a slight advantage in the use of 3D printing technology. For the surgical approach, the raters changed their choice 31% of the cases (Table 5). The data showed a slight tendency to choose medial approaches more often than lateral or posterior approaches when planning the surgical procedure with the 3D printed model. However, overall, lateral approaches were chosen much more frequently. Among the lateral approaches, the anterolateral approach was chosen more often while the extended lateral approach was chosen less frequently. Among the medial approaches, the posteromedial approach was selected more frequently after 3D-model evaluation. In addition, the posterior approaches were selected more frequently after 3D-print evaluation. When comparing the selected approaches to the approaches used in the actual treatment, the 3D-printing modality showed the highest agreement at 35% compared to CT (30%) and 3DCT (31%). While kappa and PM values slightly increased from CT to 3D printing in most subgroups, overlapping confidence intervals again indicate that these changes were not statistically significant.

**Table 3:** Interobserver agreements for the surgical approach.

Surgical approach	CT		3DCT		3D	
	PM (95% KI)	κ (95% KI)	PM (95% KI)	κ (95% KI)	PM (95% KI)	κ (95% KI)
Overall	29% (11 − 50%)	0.227 (0.227–0.277)	29% (11 − 50%)	0.220 (0.219–0.220)	33% (14 − 55%)	0.266 (0.266–0.266)
Medical Students	29% (11 − 50%)	0.184 (0.182–0.185)	28% (11 − 50%)	0.183 (0.182–0.185)	44% (24 − 68%)	0.367 (0.365–0.368)
Surgical residents	27% (11 − 50%)	0.215 (0.214–0.216)	25% (11 − 50%)	0.185 (0.184–0.185)	31% (14 − 55%)	0.242 (0.242–0.243)
Junior Surgeons	35% (17 − 59%)	0.284 (0.281–0.287)	35% (17 − 59%)	0.282 (0.279–0.284)	32% (14 − 55%)	0.260 (0.258–0.263)
Senior Surgeons	36% (17 − 59%)	0.274 80.272–0.276)	36% (17 − 59%)	0.277 (0.275–0.279)	41% (21 − 64%)	0.322 (0.320–0.324)

**Table 4:** Intraobserver agreements for the primary endpoint.

Intraobserver agreement	CT	3DCT	3D	Overall
	κ	κ	κ	κ
Treatment concept	0.40	0.41	0.45	0.35
Patient positioning	0.41	0.41	0.46	0.42
Surgical approach	0.36	0.38	0.40	0.37

**Table 5:** Change in preoperative planning after 3D-printing.

Change in preoperative planning	%
Treatment concept	5
Patient positioning	21
Surgical approach	31

### Selection of osteosynthetic plates

When the rater chose to treat the fracture with open reduction and plating, the plates selected matched the plates used in the current treatment in 63% of the cases. The percentage of matching plates was 84% for AO B fractures and 46% for AO C fractures.

### Subjective certainty

The five-point Likert scales showed a nonstatistically significant improvement in subjective confidence in the surgical approach. Categories of confident diagnostic choices (category: I feel safe & very safe) were selected 16% more often when evaluating the fracture with the 3D-printed specimen (CT 44% > 3DCT 45% > 3D-print 60%).

### Change of surgical concept and informative benefit

When directly asked whether the 3D-printed model had changed their overall surgical strategy, 36.4% of the raters responded affirmatively. Independently, rater decisions changed in individual aspects such as surgical approach (31%), patient positioning (21%), or treatment concept (5%) (Table 5).In addition, raters reported that they gained information from the 3D-printed fracture assessment in 76% of cases.

## Discussion

The most important finding of this study is that the use of 3D printing technology influenced several aspects of surgical decision making for tibial plateau fractures. While the overall match between rater choices and actual surgical treatment was moderate, 3D-printed models led to changes in surgical planning and appeared to increase subjective decision confidence across experience levels, although this effect was not statistically significant. These effects were most pronounced among less experienced raters, particularly with regard to patient positioning and surgical approach, although the observed improvements did not reach statistical significance.

Overall, the treatment concept evaluation showed low interobserver kappa values. This may be due to a paradox of high agreement but low kappa values due to the large number of raters and the influence of the prevalence of the evaluated entity, as previously described by Feinstein et al.^34^. To address this, percentage match was included as a descriptive complement to kappa, enabling a more comprehensive interpretation of interrater agreement. While not intended as a replacement for inferential statistics, PM helped to contextualize the observed agreement levels, particularly in subgroups with lower experience. The high agreement observed in the PM evaluation regarding the treatment concept may be due to the complexity of the cases, as the gold standard for such intra-articular fractures is open surgical procedure^35^. Furthermore, improvements in patient positioning and surgical approach by less experienced rater groups may be partially attributed to the newly gained haptic fracture understanding. It improves the spatial understanding of the fracture, which helps in planning the surgical procedure. More experienced surgeons may already be better in this regard, reducing the potential for improvement. These rater groups tended to have higher overall agreement values, but less improvement.

The study shows that medical students and less experienced surgeons benefit the most from the addition of 3D printing technology, as they show the greatest improvement in their assessment of concordance for preoperative measures. Wu et al. were able to show in a randomized controlled trial that the use of 3D printing is beneficial for students’ understanding of bone spatial anatomy and fractures in some anatomically complex sites^36^. This conclusion is supported by findings in similar studies of acetabular fractures or in neurosurgery^37,38^. Brouwers et al. demonstrated that 3D-printed models improved understanding, classification and surgical evaluation of acetabular fractures and found that junior residents benefited more than senior surgeons in selecting patient positioning and surgical approach when using physical models compared to CT imaging - an effect consistent with our own observations^37^. In addition, Li et al. presented a meta-analysis highlighting the educational benefits of 3D-printed models in medical training, reporting enhanced understanding of anatomy, pathology, and radiological correlation across various specialties including neurosurgery and radiology^38^.

In this study, the kappa and PM values were expected to be lower in the reliability assessment, as many raters reduce the chance of high agreement. Nevertheless, such a high number of raters ensures a high redundancy of the measured results. However, the PM analysis seemed to provide a more accurate and appropriate representation of the results. Results from comparable studies that differ from this study may be due to differences in the number of raters, cases, pretest instruction, and quality of imaging techniques. The observed numerical trends - particularly among less experienced raters - may indicate a potential benefit of 3D-printed models in improving decision-making processes in complex tibial fractures. While these effects may not directly impact clinical outcomes in inexperienced users, the educational benefit of 3D printing in surgical training is well documented and supported by our findings^36–39^.

It must be acknowledged that senior surgeons are generally accustomed to basing their surgical strategy on CT and 3DCT data alone. Nevertheless, our findings show that across all participating surgeons — including both residents and experienced specialists—76% perceived an informational benefit from the 3D-printed models. While this effect may not have led to changes in patient positioning, surgical approach, or the overall treatment concept, it suggests that 3D printing supports surgical conceptualization and may enhance the cognitive representation of complex fracture morphology. The observed trend towards higher values in subjective certainty further underlines the potential value of 3D printing, even in experienced hands.

The use of 3D printing enabled surgeons to pre-select the correct osteosynthesis plates in 63% of cases. This may be due to the variety of plate selection and positioning options available to surgeons when both compartments of the tibial plateau and the metaphysis are compromised. While many lateral split or split-depression fractures can be treated with a standard anterolateral plate, more complex patterns—particularly those involving posteromedial or bicondylar components—require individualized implant strategies. Although 3D-printed models do not reflect intraoperative limitations such as the soft tissue envelope or interference from the fibular head, they allow surgeons to better visualize fracture morphology and explore different implant configurations in advance. Correct preoperative plate selection may have the potential to reduce operative time and the number of intraoperative fluoroscopies, as demonstrated in previous studies^37,39–41^.

Another aspect of using 3D printed models in combination with osteosynthesis plates is the ability to pre-bend the plates that the surgeon plans to use. For this use, the 3D printed models would have to be sterilized. This advantage has been published several times and has a positive impact on plate fit and reduces operative time^42,43^.

Through all of this, the use of 3D printing technology is becoming more evident. This study and others suggest that fracture assessment using 3D-printed patient-specific models may enhance the preoperative planning process, particularly in training contexts^29,37,39,40^. However, statistical significance was not achieved, and observed trends must be interpreted with caution. Furthermore, the use of these specimens should always be combined with a thorough study of the CT scans, 3D volumetric reconstructions, and other conventional imaging techniques that should form the basis of any fracture evaluation^44^.

### Limitations

Although differences in kappa and PM values were visible between modalities and subgroups, statistical significance was not formally assumed due to overlapping confidence intervals and the descriptive nature of PM. Due to the large number of total ratings (*n* = 22), even small differences in agreement may have appeared statistically significant. However, the clinical relevance of these differences should be interpreted with caution. This phenomenon has been described in studies examining interrater reliability metrics in large-scale settings^34^.

Wainwright et al. suggested the existence of a fatigue factor that may decrease the rater’s perception of detail after evaluating a large number of radiographic images^45^. To overcome this problem, each rater was given the opportunity to interrupt the questionnaire at any time and continue later. However, this potential bias cannot be completely excluded. Furthermore, for ethical and radiation protection reasons, referred patients with external CT scans did not receive a second CT scan at the study site for scientific purposes. If their CT scans met the technical requirements for the 3D printing process, they were included in the study. This does not rule out the possibility that there may have been slight differences in the printed models - due to different CT scanners and scanning protocols at the primary clinic. However, all DICOM datasets were processed using a standardized segmentation and STL export workflow to ensure consistency in model geometry. Minor differences in resolution or contrast may have affected the representation of very fine fracture lines or cortical irregularities, but the overall fracture morphology was preserved in all models. A relevant impact on fracture assessment or surgical planning appears unlikely based on these differences. In addition, the plates offered to the evaluator to treat the fracture were only proximal tibia-specific plates. However, the surgeons may have used generic plates for their current treatment of the fractures, thereby reducing the likelihood of correct plate selection. This may have resulted in lower plate matching data.

One limitation of using 3D-printed models for preoperative planning is the time required for data processing, model generation, and printing. While this process can typically be completed within 24–48 h in well-equipped centers, it may not be feasible in acute trauma settings where surgical intervention is required urgently. As such, the use of 3D printing may currently be limited to elective or semi-elective procedures where adequate lead time is available. Nonetheless, with increasing availability of in-house printing infrastructure and faster production workflows, the logistical barriers are expected to diminish in the near future.

## Conclusion

In conclusion, the use of 3D-printed models supported fracture assessment and showed trends toward improved intra- and interobserver agreement regarding patient positioning and surgical approach, particularly among medical students and junior surgical residents. Experienced surgeons demonstrated more stable decision patterns, yet still reported a subjective informational benefit in the majority of cases. Additionally, the 3D-printed models enabled implant pre-selection and may have contributed to greater decision confidence, although these effects were not statistically significant. Overall, 3D printing appears to be a valuable tool for enhancing preoperative planning, especially in complex fracture cases and in training contexts.

## Data Availability

The datasets generated and analyzed during the current study are available from the corresponding author upon reasonable request. Due to legal and ethical restrictions, raw patient imaging data used for the creation of the 3D-printed fracture models cannot be publicly shared, as they contain potentially identifying information. However, all anonymized survey responses and statistical analysis results are not subject to these restrictions and can be made freely available upon reasonable request to the corresponding author for non-commercial research purposes.
